# Transactions of the Chicago Gynæcological Society

**Published:** 1880-05

**Authors:** H. Webster Jones


### Society Reports.

### ARTICLE VIII.

TRANSACTIONS OF THE CHICAGO GYNÆCOLOGICAL SOCIETY. Meeting April 16, 1880. President Prof. BYFORD in the chair. Topic, Retroversion of the Uterus. H. WEBSTER JONES, M.D., introduced the subject in the following paper:

The general adoption of the term "uterine displacement" presupposes a uterine "situs," i. e., a *normal position.*

The uterine *norme* is preserved when its axis is parallel with that of the superior strait, its fundus at or just beneath the plane of that strait, its os an inch and a half to two inches above the floor of pelvis (or tip of coccyx), the cervix distant from the third sacral bone an inch and a half, and from the sub-pubic ligament two and a half inches.

Deviations from these relations are common, and, within certain limits, normal.

When normal, they are transitory and due to variable distentions of the bladder, of the rectum, of the abdominal viscera, or to active erectility of the tissues of or surrounding and connected with the uterus.

When abnormal, they are more or less persistent, depend upon vicious states of the uterus, or of the organs and tissues with which it is related, or yet may be purely mechanical, and in either case, competent to exercise morbid influences over nerves and nerve centers, perverting nutrition, sensation and functional offices in a remarkable degree.

When a section of the pelvic contents is projected upon a plain surface in accordance with the above postulate, the uterine axis will be found meeting that of the vagina at or about a right angle, which becomes acute as the uterus descends to impinge upon the pelvic floor, and obtuse only as the cervix slides forward along its inclined plane.

When in this process, however induced, the uterine passes beyond the vertical axis of the body, retroversion may be said to have commenced.

It may attain any degree of divergence from the *norme* compatible with the dimensions of the pelvic cavity; in general terms, from one to eighty degrees from a perpendicular, or sixty to one hundred and forty from the *norme*.

The uterus projecting downward through a plane called the "pelvic roof," which intersects the sub-pubic ligament and the junction of the third and fourth sacral bones, is sustained by cellulo-muscular processes—continuations of its own structure (Rouget), some of which are called ligaments, whose lines of force radiate in the plane mentioned from the cervix toward the sacrum, sacro-iliac junctures, and walls of the pelvic cavity like a fan whose handle the vagina well represents. It is upon the normal antagonisms of all these forces, vagina, utero-sacral and lateral ligaments, that the *situs* of the uterus depends, the vesico-uterine folds the round ligaments and anterior muscular structures of the perineum, being adjuvants worthy of mention.

It needs only a general relaxation of these supports, whether of local or systemic origin, to permit lapse and prolapse of the uterus. And when persistent infringement of the cervix upon the perineal structures obtains, retroversion is sure to follow.

On the other hand, given a loss of balance of antagonisms due to pathological contraction or relaxation of connective tissue, or a localized lessening of erectility due to embolism of curling arteries, or thrombus of venous trabeculæ, and we have divergence of the uterine axis. (Patten.)

*More or less flexure of the uterus obtains in every case of retroversion.*

Among predisposing causes of retroversion are neurasthenic muscular inertia and local congestions, constipation, intestinal and vesical distention, abdominal compression, subinvolution, chronic inflammation of the uterus or of its supporting tissues, and pelvic tumors.

Exciting causes include violent and sudden muscular efforts, falling, dancing and riding on horseback, especially when the bladder is meantime distended, and last, not least, the incautious use of the speculum.

Treatment may be successful in removing the cause, yet if the displacement itself be ignored, the patient may not only be unrelieved, but become, therefore, the subject of erosions and ulcerations, leucorrhœa, dysmenorrhœa, menorrhagia, metrorrhagia, sterility, dyspareunia, rectal and vesical irritability, neurasthenia, and many reflex and functional nervous aberrations.

Retroversion becomes important soon after puberty, and diminishes in importance after the climacteric.

As illustrations of the manner of demonstration of this truth to the mind of the speaker, he selects four cases, out of a multitude of instances similar in character.

*Case I.*—The wife of a railway conductor, who had miscarried two years before, related the following story :

Six months after her miscarriage, she was, while sweeping, seized with a violent pain in the epigastrium, which she described as an "unbearable twisting" of the subjacent organ, lasting for a half hour, when she became insensible.

The paroxysms at first recurred whenever she swept, and after a few weeks, whenever she walked the distance of half a mile. All treatment proving unavailing, she was brought to the city for advice. The uterus was found completely retroverted, and enlarged to the measurement of four inches. There was no subjective symptom which pointed to this explanation. But this patient never experienced another paroxysm after the uterus was reinstated.

*Case II.*—The wife of a civil engineer engaged in building a neighboring line of railway, sent for me, as she could not endure the journey of ten miles to the city. Her only exercise was taken in a sedan-chair, when borne by the laborers to a shady spot, whence she could view the progress of the work, and have the society of her husband. She was found to have complete retroversion, extensive erosion, exhaustive leucorrhœa, wretched appetite and digestion, and was hopelessly dejected.

Two weeks were allowed me for a cure. I demanded her removal to the city, and visited her daily, applying emollients and stimulants, and finally adjusting a pessary.

She disappeared suddenly thereafter, and I knew nothing more of the case for five years, when she visited me with an apology for the abruptness of her departure, and the non-payment of fees, and desired me to remove the pessary, which she had never disturbed in the interval.

The uterus was found *in situ*, sound in every respect, and the general condition of the patient was so excellent that I could never have recognized her. She averred that her improvement was so rapid after the ten days of treatment mentioned, that she followed her husband into the woods of Wisconsin, and soon was able to take charge of the cooking and housing of the hundred men then under his orders. She had followed his fortunes ever since, never caring to remove the pessary, to which she attributed her cure. It came out as clean and nearly as polished as when introduced.

*Case III.*—A lady of twenty-five years began to have menorrhagia soon after the birth of her only child. The drain, in two years' time, became constant and irremediable by any of the usual hæmostatics. Anæmia was excessive, and the general condition so alarming that a consultation was had, and curetting performed, but without success. Tampons gave the only palliation, and these did not prevent a persistent oozing of blood. I saw this lady a month after the operation alluded to, and restored to its place an enlarged and retroverted uterus. In twenty-five hours the discharge became pale and watery, and in a week no vestige of it remained. She has since, for about nine months, menstruated regularly and normally, though the subject of constant albuminuria. (Since dead.)

*Case IV.*—A lady had been for six months the victim of that train of symptoms denominated "neurasthenia." She had anorexia, insomnia, palpitations, constant backache, constipation, colonic catarrh, indigestion, and at times a mental paresis and childish apprehension that was pitiable to see. For exercise she was lifted from her bed to a carriage, and driven for hours every day, and had frequent massage. Every resource that science could suggest had been applied in her treatment, but without success. No examination of the uterus had been made.

At that interview the uterus was found retroverted, but *not in any other way* abnormal. Its reposition was followed by immediate relief to the backache, and a gradual amelioration of all the symptoms, until in two months' time she considered herself perfectly restored.

The following propositions are presented for discussion :

1. The uterus has a normal situs, with normal deviations which are transitory.

2. Deviations which are constant, or which exceed the natural powers to obviate, are morbid and morbific.

3. Retroversion is the most common, most pronounced and most vicious of uterine displacements.

4. It is often present without subjective consciousness on the part of patient, nevertheless being *pars magna*, if not *fons et origo mali.*

DISCUSSION.

DR. NELSON spoke of the difficulty of establishing a normal situs for the uterus. He called that a case of retroversion when the axis of the uterus was parallel with the axis of the vagina. The speaker mentioned a case which had occurred under his observation, where the uterus was found in a state of retroversion at one examination, and a few days later was found in a condition of anterior displacement, only to be found again retroverted.

Dr. FITCH had also encountered cases like that just spoken of by Dr. Nelson, and thought it not uncommon for a uterus to be displaced in opposite directions within a period of some days. On the whole, the speaker accepted the propositions enunciated by Dr. Jones. Dr. Fitch said that the effects of uterine displacements were variable, and he believed it possible that a woman may have marked retroversion of the uterus with absolutely no effect.

DR. ROLER believed that the uterus had a normal situs but it was only when some morbid conditions occurred referable to the unusual position of the organ, that the situs should be called abnormal.

DR. MILLER : Dr. Jones has presented his subject in the most exact manner possible. It is as concise as exact. Having predicated his conclusions on geometrical and mathematical science, he gives no latitude for imagination or discursive conversation. We are held to the consideration of lines, angles and figures, and from these there seems no way of escape. I concur in the description of the anatomical situation and relations given by the essayist. While retroversion may take place in varying degrees, the extent of the displacement cannot be taken as a measure of its pathological significance. The factors are so varied, that in one case the initial link in the chain of morbid effects will be merely mechanical—in another congestive—and in a third neuralgic—and the effects may be reflected to different and distant parts of the system.

DR. SUTTON (present by invitation) said that he regarded any position of the uterus a normal one, which produced no abnormal effects upon the woman, and which did not in any degree interfere with the functions of the organ. He would ask Dr. Fitch, if it were possible for a woman to suffer an abnormal position of the uterus with absolutely no symptoms, what there was about such a subject which led to a vaginal examination and discovery of the displacement.

DR. SAWYER believed that the uterus had a normal situs, but that that situs varied in different subjects; he thought that females were subject to as marked differences in those parts as in their facial features. The fundus moves through an arc from before backward; the extremities of this arc vary according to the condition of plenitude or vacuity of the bladder and rectum in the particular individual. Inasmuch, however, as the distension of the bladder and rectum is a factor which varies according to the habit of the woman, this fundal arc may be of a length which is physiological for one person and abnormal for another.

The speaker said further that the altitude of the organ was also subject to variations. Dr. Jones had stated that the cervical tip of the organ should be an inch or an inch and a half above the pelvic floor; these limits the speaker thought too narrow and greatly dependent upon one very important condition. The columns of the vagina are looked upon as the chief antagonists of the gravity of the uterus. Now, the angle at which these supports meet the uterus, in a word, the efficiency of the support itself, depends much upon the size of the perineal body. It is greater in women who have not borne children; consequently the uterus is normally higher in these persons. The perineal body with the external genitalia seem to shrink, as if subjected to a sort of involution, after child-bearing, and in these persons, on the contrary, the uterus is normally lower. For these reasons the normal situs of the uterus must be described for each individual, and the diagnosis of the slight aberrations from this situs must be founded upon the effects produced by them. The speaker admitted, however, that the uterus, when in the situs ascribed as normal by the essayist, does not produce any abnormal effects.

THE PRESIDENT said that within his recollection the sentiments and practice of the profession in this country had undergone two or three very considerable changes in reference to the importance of misplacements. In the early part of the century the doctrines of Prof. Hodge were the guide to practice in gynæcology. The displacements of the uterus were regarded as the principal causes of the conditions now known as hystero-neurosis or orphaso-neurosis, whichever we choose to call them. After this the writings of Prof. Meigs and Dr. Bennet created a revolution in professional opinion, and to chronic inflammation and ulceration was attributed all the blame for the sufferings of women. The pendulum of professional opinion has swung back to it and has not settled down to the proper medium. Our experience has taught us that between extremes we can generally find the truth. We seldom find displaced uteri healthy in other respects and the question is whether the displacements or the attendant diseases are the real *fons et origo* of the general symptoms. At any rate the speaker could not doubt that the best practice consisted in correcting all the abnormal states of that organ. And while he willingly endorsed all the propositions advanced so clearly and distinctly in Dr. Jones' paper, he might not treat the cases met with in practice exactly as that accomplished practitioner would.

DR. JONES: In closing the discussion, the essayist said, by way of explanation of his "geometry" of the pelvis, that it was an instructive lesson to attempt to draw upon paper the uterus or its connections in accordance with the dicta of authority.

Perhaps the most unanimous point was the length of the uterus, and next, the position of fundus, just below the pelvic brim, and last, the parallelism of the uterine axis with that of the superior strait. Given "approximately" these points, and with a "standard" pelvis, the "geometry" described would surely follow.

All that he claimed for retroversion was that no gynæcologist could afford to ignore it, and that its mechanical treatment was, like that of hernia, one horn of a dilemma, the advantages and disadvantages of which it well paid to study.

